# Molecular gut content analysis of different spider body parts

**DOI:** 10.1371/journal.pone.0196589

**Published:** 2018-05-30

**Authors:** Nuria Macías-Hernández, Kacie Athey, Vanina Tonzo, Owen S. Wangensteen, Miquel Arnedo, James D. Harwood

**Affiliations:** 1 Department of Entomology, University of Kentucky, Lexington, Kentucky, United States of America; 2 Department of Evolutionary Biology, Ecology and Environmental Sciences, Universitat de Barcelona, Barcelona, Spain; 3 Biodiversity Research Institute (IRBio), Universitat de Barcelona, Barcelona, Spain; 4 Norwegian College of Fishery Science, UiT The Arctic University of Norway, Tromsø, Norway; 5 College of Plant Health and Medicine, Qingdao Agricultural University, Qingdao, Shandong, China; University of Richmond, UNITED STATES

## Abstract

Molecular gut-content analysis has revolutionized the study of food webs and feeding interactions, allowing the detection of prey DNA within the gut of many organisms. However, successful prey detection is a challenging procedure in which many factors affect every step, starting from the DNA extraction process. Spiders are liquid feeders with branched gut diverticula extending into their legs and throughout the prosoma, thus digestion takes places in different parts of the body and simple gut dissection is not possible. In this study, we investigated differences in prey detectability in DNA extracts from different parts of the spider´s body: legs, prosoma and opisthosoma, using prey-specific PCR and metabarcoding approaches. We performed feeding trials with the woodlouse hunter spider *Dysdera verneaui* Simon, 1883 (Dysderidae) to estimate the time at which prey DNA is detectable within the predator after feeding. Although we found that all parts of the spider body are suitable for gut-content analysis when using prey-specific PCR approach, results based on metabarcoding suggested the opisthosoma is optimal for detection of predation in spiders because it contained the highest concentration of prey DNA for longer post feeding periods. Other spiders may show different results compared to *D*. *verneaui*, but given similarities in the physiology and digestion in different families, it is reasonable to assume this to be common across species and this approach having broad utility across spiders.

## Introduction

The use of DNA-based methods to study food webs and feeding interactions under natural conditions has revolutionized dietary analysis in a variety of ecosystems, including marine environments [1], agroecosystems, [2–5], forests [6], and soils [7]. The detection of prey DNA has revealed a broad range of trophic relationships in nature (reviewed in [8–11], with a multitude of implications for conservation biology and management [12]. DNA-based methods facilitate prey identification in the absence of hard prey remains, as is usual for many invertebrate systems. Compared to other methodologies (e.g. morphological identification of prey remnants in the stomach or faecal samples, enzyme-linked immunosorbent assays (ELISA) techniques, or using monoclonal antibodies, among others), DNA-based prey assays can be developed faster, allow simultaneous screening for multiple prey items and offer a greater taxonomic prey resolution, although DNA prey detectability may span shorter periods of time [10, 13]. For vertebrates [14–17] and some invertebrates [18, 19], molecular methods can also be applied using non-invasive methods by analysing regurgitates or faecal samples. Although many predaceous arthropods are liquid feeders or employ extra-oral digestion, usually requiring post-mortem analysis, recent studies have demonstrated the viability of using faecal analysis for prey identification in spiders [20]. However, typically gut-content analysis in invertebrates requires killing the animal and either dissecting their gut (e.g. in beetles), or selecting body parts for DNA extraction, depending on the size and identity of the focal predator [8].

A multitude of inexpensive chemicals (e.g. sodium dodecyl sulfate, SDS; or cetyltrimethyl ammonium bromide, CTAB) [21] have been adopted for gut analysis, although commercial extraction kits (e.g. Qiagen Blood and Tissue kits, Qiagen Inc, Valencia California, USA) are popular for gut content analysis because they are fast and easy to use, and more effective at amplifying gut contents [22]. However, extraction kits limit the amount of predator tissue that can be extracted. For instance, samples when using DNeasy^®^ kits cannot exceed 25 mg in mass. While for small sized predators the whole specimen can be used for DNA extraction, large predatory arthropods may require gut dissection [6, 23] and predators with branching digestive tracts that occupy most of the body represent additional challenges when deciding what section of the body is most useful for detecting prey.

Spiders are liquid feeders utilizing a process of extra-oral digestion for prey consumption. Following ingestion of liquefied material, their midgut branches into highly complex diverticula extending throughout the prosoma and into their legs [24, 25]. Consequently, digestion takes place in many different parts of the body and dissecting the whole gut is near to impossible. When performing molecular gut-content analyses, spiders need to be either small-sized (for total body extraction), split into body parts prior to extraction or be homogenized with a subset used for DNA extraction. Most studies have focused on small-sized spider families such as the Linyphiidae [26, 27], small Lycosidae [20, 28, 29], Theridiidae, Salticidae [30], small Tetragnathidae [31, 32] and Oxyopidae [33], in which DNA extraction was made either by homogenizing the whole spider or by crushing the abdomen. Although many studies have used spiders for molecular gut-content analysis, almost none have used medium to large body size spiders (but see Schmidt *et al*. [2]).

To investigate the detectability of prey DNA within the gut of a medium sized spider (15–20 mm; weight: males 40–60 mg, females 50–100 mg), we use the woodlouse hunter spider *Dysdera verneaui* Simon, 1883 (Araneae: Dysderidae). *Dysdera* is a highly speciose genus distributed throughout the Mediterranean basin, with the exception of the cosmopolitan species *D*. *crocata* [34]. It has also colonised some oceanic archipelagos, such as the Canary Islands, where it has undergone a major process of local diversification (approximately 50 endemic species occur throughout this archipelago [35]). They are nocturnal wandering hunters that prefer humid and dark ground habitats and during daytime find shelter in silk retreats under rocks, trunks and tree barks. Unlike other spider genera, *Dysdera* shows a remarkable diversity of body sizes and mouthpart shapes [36, 37]. These differences have been related to both trophic specialisation (some species are generalists while others are woodlice feeder specialists) [38] and the prey capture strategies used to feed on woodlice [39]. The evolution of different levels of prey specialisation and preference has been identified as one of the major drivers of *Dysdera* diversification in the Canaries [40]. Thus, *Dysdera* offers an invaluable model for applying molecular gut analysis to decipher their diet in natural habitats.

Here, we combine feeding experiments with two methods of molecular prey detection, namely prey-specific PCR and metabarcoding approaches, to investigate molecular detectability of diet in medium-sized predators that exhibit extra-oral digestion. Specifically, we tested the differences on prey detectability in the spider *D*. *verneaui* using DNA extracts from different body parts (i.e., legs, prosoma and opisthosoma) and characterized the time of prey detectability after consumption. Based on these results, we provide suggestions for optimizing detection of prey DNA when using molecular gut content analysis in medium sized spiders.

## Material and methods

The Cabildo of Tenerife authorized the collecting permits for the protected natural areas.

### Specimen collection

Eighty specimens of *D*. *verneaui* were collected at the laurel forest (dominated by *Laurus novocanariensis*, *Ilex canariensis*, and *Persea indica* tree species) of Anaga in Tenerife (28.535600 N, 16.298810 W) (Canary Islands, Spain) during 2013. Spiders were hand-collected by searching under stones and logs, scraping soil and at rocky embankments. Each individual was placed into a separate 1.5 mL vials. Individuals of the potential target prey, the woodlice, *Eluma caelata* Miers, 1877 (Isopoda: Armadillidiidae), were collected at the same site. Additionally, non-target taxa found in the habitat were collected for construction of a DNA barcode reference library and to test for natural predation. Larvae of *Tenebrio molitor* Linnaeus, 1758 (Coleoptera: Tenebrionidae) reared in laboratory colonies were used as chaser prey.

### Feeding trials

Feeding trials were conducted to determine the detectability half-life (after Greenstone & Hunt [41], reviewed by Greenstone [42]) of prey DNA in the gut of *D*. *verneaui*. These approaches were also used to test for differences in detectability between alternative body parts, namely legs, prosoma and opisthosoma. Predators were maintained on a 14:10 h light:dark cycle at room temperature, in individual plastic Petri dishes (55 mm) containing wet filter paper laid at the dish base. All predators were deprived of food for two weeks prior to starting the experiments. After this period, each spider was offered one specimen of the target prey (*E*. *caelata*) in the dark because *D*. *verneaui* are nocturnal wandering predators.

Spiders were observed to feed and the time of completion of the entire woodlouse was taken as reference time zero (t = 0). At this time, eight specimens were immediately transferred to individual autoclaved 1.5 mL microcentrifuge tubes containing 95% ethanol and subsequently stored at -20°C. All remaining predators were provided with larvae of *T*. *molitor* as a ‘chaser prey’ and maintained as above for 2, 4, 8, 18, 24, 48, 72, 96 or 120 h after feeding (n = 8 killed as described above at each time point). All samples were stored at -20°C until DNA extraction.

### Spider dissections

**Fig 1:**
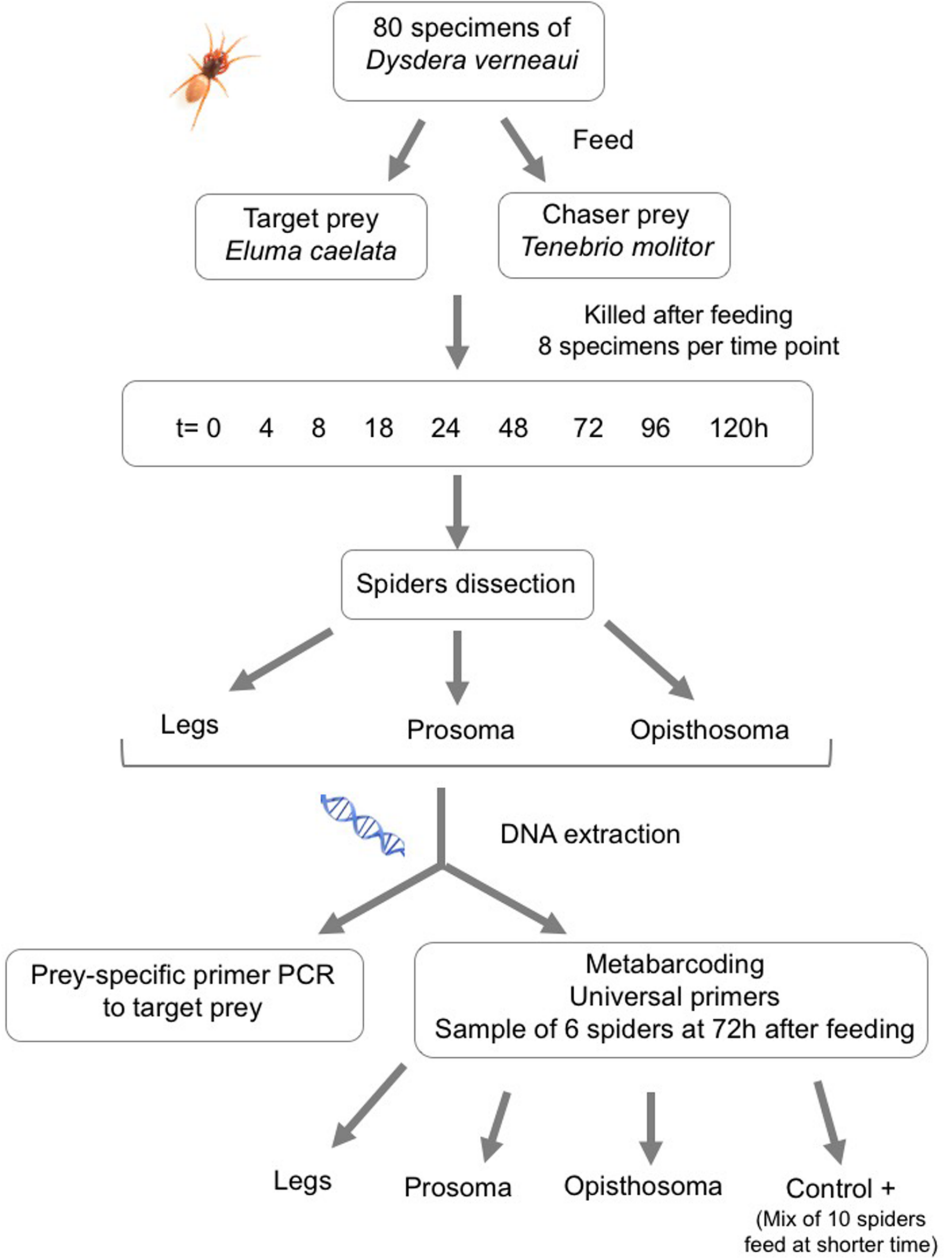
Diagram showing the experimental design and methodology used in the study.

To investigate prey DNA detection in different spider body parts, specimens of *D*. *verneaui* were dissected into three parts: i) all the legs (excluding metatarsus and tarsus), ii) the prosoma, and iii) the opisthosoma. Dissection was conducted using forceps and scissors, flame sterilized after each dissection to prevent cross contamination. The dissected parts were then subjected to DNA extraction separately. See Fig 1 for more details.

### DNA extraction, primer design, PCR amplification and sequencing

To obtain sequences for primer design, total DNA was extracted from legs of target prey, *E*. *caelata*, and non-target specimens (S1 Table) using QIAGEN DNeasy Blood and Tissue Kits (QIAGEN Inc., Valencia, California, USA) following the manufacturer’s guidelines. We targeted the animal DNA barcode, the mitochondrial gene coding Cytochrome c oxidase subunit I, hereafter referred as COI, as the molecular marker. Due to difficulties in amplifying some species, DNA was amplified using a combination of several universal COI primers: LCO22me and Hco700dy [43], Fol-degen-F and Fol-degen-R [44], or CI-J-2183 and L2-N-3014 [45] (Table 1). PCRs (50 μL) consisted of 1X Takara buffer (Takara Bio Inc., Shiga, Japan), 0.2 mM of each dNTP, 0.2 mM of each primer, 1.25 U Takara Ex Taq^™^, Bovine serum albumin (BSA) (20mg/ml) (Promega Co., Madison, Wisconsin, USA), and template DNA (4 μL of total DNA, 1/10 diluted). PCRs were carried out in Bio-Rad PTC-200 and C1000 thermal cyclers (Bio-Rad Laboratories, Hercules, California, USA). The PCR cycling protocol was 94°C for 3 min followed by 40 cycles of 94°C for 45 s, 40°C for 45 s, 72°C for 45 s, and a final extension of 72°C for 10 min. Reaction success was determined by electrophoresis of 10 μl of PCR on a 2% SeaKem agarose gel (Lonza, Rockland, Maine, USA), pre-stained with 1X Gel Red^™^ nucleic acid gel stain (Biotium, Hayward, California, USA). DNA sequencing was undertaken at Advanced Genomics Technologies Center (University of Kentucky, Lexington, Kentucky, USA).

**Table 1:** List of primers used in the present study: (1–6): *cox1* primers used to amplify target and non-target prey; (7–8): New specific primers designed to amplify *Eluma caelata*; and (9–10): Primers used to amplify the *cox1* region for the metabarcoding analyses.

Primers	Name	Sequence (5'- 3')	Reference
1	Lco22me	GGTCAACAAATCATAAAGATATTGG	Walker *et al*., 2006
2	Hco700dy	TCAGGGTGACCAAAAAATCA	Walker *et al*., 2006
3	Fol-degen-F	TCNACNAAYCAYAARRAYATYGG	Yu *et al*., 2012
4	Fol-degen-R	TANACYTCNGGRTGNCCRAARAAYCA	Yu *et al*., 2012
5	CI-J-2183	CAACATTTATTTTGATTTTTTGG	Simon *et al*., 1994
6	L2-N-3014	TCCAATGCACTAATCTGCCATATTA	Simon *et al*., 1994
7	Elu-F306	GAGGGTTGGTTGAAAGTGGC	(Generated in this study)
8	Elu2-R510	AAAGGAACTCGATCTATTTTA	(Generated in this study)
9	mlCOIintF-XT	GGWACWRGWTGRACWITITAYCCYCC	Wangensteen *et al*., 2018
10	jgHCO2198	TAIACYTCIGGRTGICCRAARAAYCA	Geller *et al*., 2013

Resulting COI sequences were edited and assembled using Geneious 7.1.9. (Biomatters Ltd., Auckland, New Zealand) [46], aligned using MUSCLE [47] and visually inspected using BioEdit 7.0.0 (Isis Pharmaceuticals Inc., Carlsbad, California, USA) [48]. Primer conditions were checked using PRIMER 3 [49]. We designed a specific fragment to amplify a 205 bp amplicon in the target prey *E*. *caelata*: Elu-F306: GAGGGTTGGTTGAAAGTGGC and Elu2-R510: AAAGGAACTCGATCTATTTTA. The alignment used for the specific primer design contained a mixture of sequences generated in this study and sequences downloaded from GenBank (see S1 Table for accession numbers).

Gradient PCRs were performed to adjust optimal PCR conditions for the primer pair Elu-F306 and Elu2-R510. Amplification of the gut-content of *D*. *verneaui* from the feeding trials was obtained using the QIAGEN multiplex kit, which has been used successfully in previous gut content studies with spiders [50] and herbivorous insects [51]. The multiplex kit is reported to overcome the effect of PCR inhibitors in the predator [52, 53] that was most likely responsible for the non-successful amplification of the gut-content of *D*. *verneaui* using the same PCR conditions described above. Each 10 μL multiplex PCR contained 2 μL of predator DNA, 5 μL of master mix, 0.5 μL of each primer (10 μM), 1.5 μL of Q-solution, 0.2 μL of BSA, and RNase-Free water to adjust the volume. The PCR cycling conditions were as follows: 15 min at 95°C followed by 35 cycles of 94°C for 30 s, 59°C for 90 s, 72°C for 60 s, with a final extension step of 72°C for 10 min. Each PCR set contained two positive controls (*E*. *caelata* and *D*. *verneaui* fed with *E*. *caelata* at t = 0) to assess reaction success, and two negative controls (no template DNA and DNA from *D*. *verneaui* tarsus) to check for cross amplification and contamination. All predators showing PCR product of expected size were scored as positive. All samples scoring negative for prey DNA were re-assayed again to check for false negative results and, if necessary, amplified using COI general primers (Table 1) to test for extraction success. To further confirm amplification success, eight PCR products of positive amplifications were sequenced to confirm the identification of prey DNA. Primers were tested against 80 non-targets to determine specificity (S2 Table).

### Metabarcoding

A metabarcoding approach was used to determine the number of prey sequences amplified from the gut of spiders at the longest time after feeding (t = 72 h) in which six specimens tested positive by PCR for *E*. *caelata*. Each of the six spiders fixed at 72 h after feeding was separated into three samples, corresponding to the three dissected body parts (legs, prosoma and opisthosoma) and analysed through metabarcoding. All the spiders had eaten both the target prey and the chaser prey (*T*. *molitor*). In order to test the ability of the universal metabarcoding primers to amplify the target prey (*E*. *caelata*), we included a positive control sample, which consisted in a mixture of 10 spiders (using different body parts) that were fed at shorter periods of time (t = 0–48 h) and tested positive in the PCR with specific primers.

DNA concentration of each extraction was determined using Qubit fluorometric quantitation (dsDNA HS Assay Kit, Thermo Fisher Scientific, Waltham, Massachusetts, USA) to adjust the final concentration of DNA in the sample to 10 μg/μL. DNA was amplified in a single-PCR step for the DNA barcode region using the universal primers mlCOIintF-XT [54] (modified from the mlCOIintF primer of [55]) and jgHCO2198 [56] producing a 313 bp amplicon. Each primer pair included an 8 bp sample tag (the same tag in the forward and reverse primers) and a tail of 2–3 random Ns in the 5' end for increasing sequence variability of the library [57]. Each 20 μL PCR reaction contained 2 μL of predator DNA (mixed DNA from 6 spider individuals in equimolar amounts), 10 μL of AmpliTaq Gold 360 Master Mix (Thermo Fisher Scientific), 1 μL of each tagged primer (5 μM), 0.16 μL of BSA, and DNase-Free water to adjust the volume. The PCR cycling protocol was 95°C for 10 min followed by 35 cycles of 94°C for 1 min, 45°C for 1 min, 72°C for 1 min, and a final extension of 72°C for 5 min. Tagged amplicons were pooled and purified using the MiniElute PCR Purification Kit (Qiagen). Illumina adapters and a library tag were added using the NEXTflex PCR-free DNA sequencing kit (Bioo Scientific, Austin, Texas, USA) and the library was sequenced in an Illumina MiSeq with a V2 2x250 bp paired-end partial run at the University of Salford, UK. The samples for this study were included in a multiplexed MiSeq run with a total of 86 samples, which explains the values for the sequencing depth of our results.

The metabarcoding pipeline was based in the OBITools suite [58]. After checking the quality of the reads with FastQC, paired-end reads were aligned using *illuminapairedend* and only aligned reads with quality score >40 were kept. The aligned dataset was demultiplexed and the primer sequences were removed using *ngsfilter*. A length filter (*obigrep*, 306–320 bp) was applied and reads containing ambiguous bases were removed. The reads were then dereplicated using *obiuniq* and the *uchime-denovo* algorithm implemented in *vsearch* [59] was used to remove chimeric sequences [60]. Molecular Operational Taxonomic Units (MOTUs) were delimited using the Bayesian clustering algorithm implemented in CROP [61] using the parameter values l = 1.5 and u = 2.5 [54]. Taxonomic assignment of the representative sequences for each MOTU was performed using the *ecotag* algorithm [58]. For the taxonomic assignment, we built a reference database using sequences retrieved by *in silico* PCR against release R117 of the EMBL-EBI database using *ecoPCR* [62]. New sequences obtained for our species of interest were added to this reference database. This combined reference database is publicly available from Mendeley Data [63]. The final refining of the dataset included taxonomic clustering of MOTUs assigned to the same species and abundance renormalization for removing false positive results [57]. This abundance renormalization procedure is based on calculating the cumulative frequencies of relative abundances of each MOTUs in every multiplexed sample, and equalling to zero the values of those samples whose cumulative frequency is < 1%. This procedure was applied to the whole multiplexed library of samples that were analysed together in the MiSeq run, and it is necessary for removing false positives resulting from random tag switching [64]. After this step, we checked that this correction did not affect the results of abundances for both studied prey items (in the sense of removing any true positive result of both prey items from the body part samples).

### Statistical analyses

The rate of decay of prey DNA (*E*. *caelata*) within the guts of spiders was calculated using the positive scores of PCR amplification with specific target-prey primers of each spider´s body part separately. Detectability half-lives of each body part tested (legs, prosoma and opisthosoma) was calculated using probit analysis, and Chi-square (*X*^2^) test were used to determine how well a probit model fit the data. The results of the three body parts were compared using the recommended 83% fiducial confidence limits [65]. All analyses were performed in SAS 9.4, (SAS Institute, Cary, North Carolina, USA).

## Results

### Spider´s body part detection and DNA decay rates

Sequences of *E*. *caelata* matched those on GenBank with 100% identity. The primers designed in the present study were specific to the target prey with no cross reactivity with 80 non-target taxa (S2 Table).

Results of target prey amplification by PCR for each body part (legs, prosoma and opisthosoma) is shown in Table 2. Probit analyses of the feeding trials showed that median detection time (MDT) of the three body parts tested overlap in 83 h (see Table 3). Decay rate curves of *E*. *caelata* DNA within the guts of predators of the three body parts is shown in S1 Fig.

**Table 2:** Detection of target prey when using prey-specific PCR approach for each body part tested (legs, prosoma and opisthosoma).

Time after feeding (h)										
	0	2	4	8	18	24	48	72	96	120
**Detection (Legs)**	7	7	8	8	6	7	7	6	2	2
**Detection (Prosoma)**	7	6	8	8	6	7	7	6	2	2
**Detection (Opisthosoma)**	8	7	8	8	6	6	6	6	2	2

**Table 3:** DNA detectability half-life of the different body parts tested calculated with probit models, and 83% fiducial confidence limits calculated to compared half-lives.

Body Part	Half Life (h)	83% fiducial confidence limits		Chi-Square	P-value
**Legs**	83.6	53.7	116.3	5.62	0.0177
**Prosoma**	83.9	52.1	126.5	5.56	0.0183
**Opistosoma**	83.2	52.2	114.4	5.55	0.0185

### Metabarcoding results

**Fig 2:**
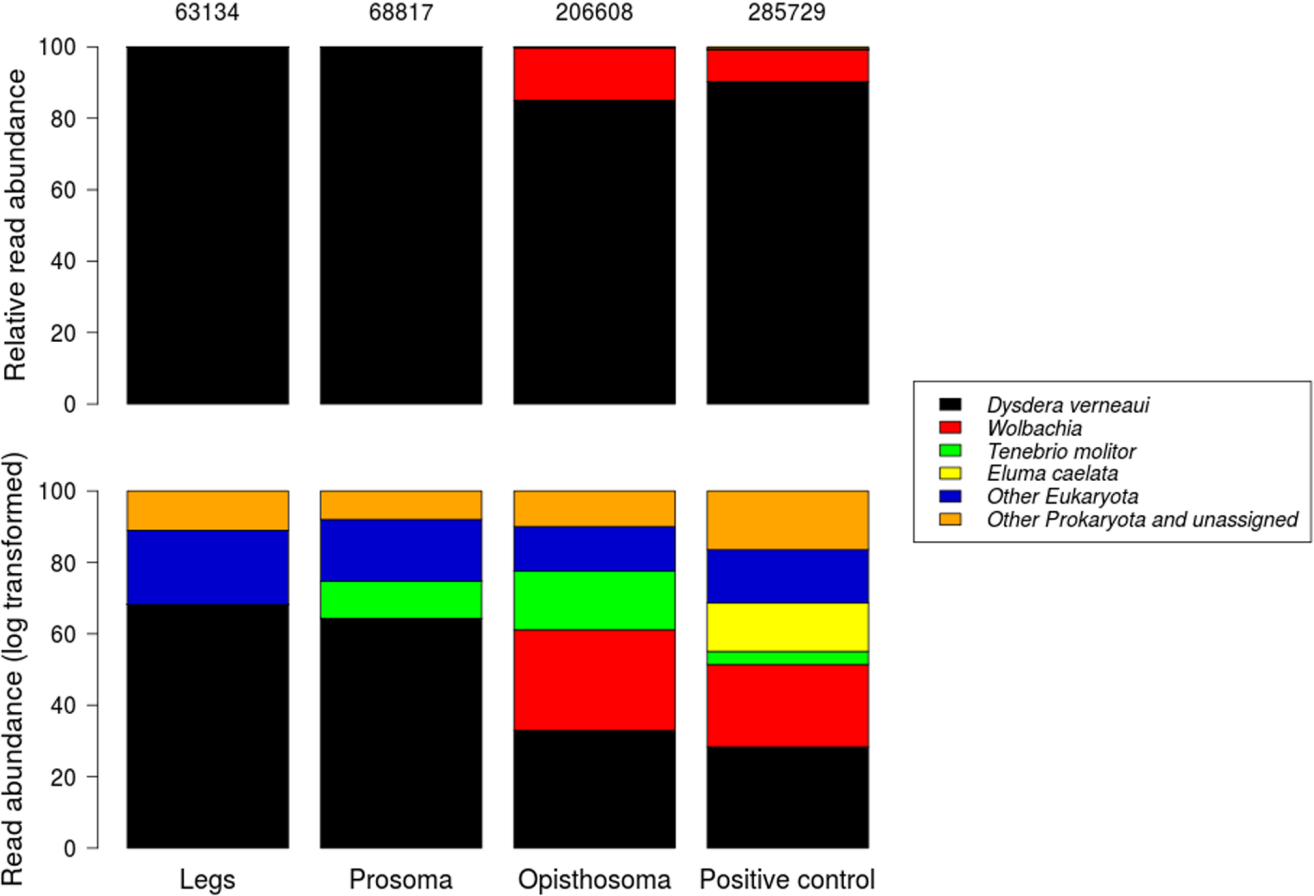
Abundance of reads of detected MOTUs in different body parts of *D*. *verneaui* 72 hours after feeding and in a positive control (a mix of 10 spiders recently fed). The total number of reads obtained for each sample (after quality control) is shown above the bar plots. Relative abundance of reads (above) and relative abundance of log-transformed reads (below) are shown, for a better visualization of MOTUs detected in low abundances.

The Illumina sequencing generated 339,385 reads after quality control for the three samples analysed (legs, prosoma and opisthosoma of *D*. *verneaui*). The number of unique sequences in these samples was 75,265, which were clustered into 443 different MOTUs by Bayesian clustering and collapsed into a final dataset of 144 MOTUs by taxonomic clustering. After abundance renormalization, this final dataset included 338,559 reads. On the other hand, the positive control sample generated 285,729 final reads. A graphical summary of read abundance of items detected in the samples is shown in Fig 2.

In all samples, most reads belonged to *D*. *verneaui* DNA (90.85% of the three samples and 90.3% of the positive control), followed by 8.97% of the reads (8.85% of the control) that were assigned (with 94.9% sequence identity) to a *Wolbachia* endosymbiont specifically localized in the opisthosoma and positive control samples (after abundance renormalization for removing false positives). In the control sample a total of 387 reads (0.13%) were assigned to the target prey *E*. *caelata*. In the three analysed body parts (legs, prosoma and opisthosoma) a total of 411 reads (0.12%) were assigned to *T*. *molitor* (5 reads in the prosoma sample and 406 reads in the opisthosoma), whereas *E*. *caelata* was not detected in any of these samples.

## Discussion

This study compared the results of a traditional PCR approach with the metabarcoding technology to identify optimal body part locations of a spider that were most suitable for amplifying partially digested prey DNA. Using a PCR approach with specific primers for a target prey, we demonstrated that all parts of the spider body (legs, prosoma and opisthosoma) are equally suitable to detect consumed prey, regardless of the different after-feeding periods tested. Traditional PCR is not quantitative and hence is unable to measure the amount of DNA contained on each sample. Alternatively, more refined techniques such as qPCR [66–69] or post-PCR visualization using a capillary electrophoresis system (e.g. [29, 67, 70]) could be utilized to achieve a more precise quantification of which body part contains more target prey DNA.

Instead, here we used a metabarcoding approach as a quantitative method. Generally, in biodiversity assessment or dietary studies, metabarcoding is not considered a quantitative method because prey amplification with universal primers might be biased toward certain taxonomic groups and many factors such as annealing temperature and PCR cycles may affect the amplification efficiency and final success [71–73]. Although with some caution because nothing is known about the digestion rate in the different spider body compartments, we propose that in this study, the number of reads obtained in the metabarcoding analyses can be used to quantify the relative abundance of prey remains in the different body parts of the same specimen, because the putative biases in the PCR can apply similarly to the three samples analysed.

Comparison of the metabarcoding analyses revealed differences in the number of prey reads (of *T*. *molitor*) detected in the different body parts tested, with the opisthosoma being the tissue containing the highest number of reads (406) and hence the best alternative for amplifying gut content after long post feeding periods. These results are in contrast to the results from PCR using specific primers, which suggested no differences among the spider body parts, but in agreement with other studies also using metabarcoding [74].

Prey DNA in the gut of a predator is highly degraded and at lower concentration than predator DNA [75] making it more difficult to amplify. Another complication is that the high concentration of predator DNA in the whole-body extracts may inhibit prey detectability [53, 76]. Although there are several methods to enhance PCR amplification and reduce the action of PCR inhibitors, such as adding an amplification facilitator (e.g. bovine serum albumin) [52] or purifying DNA extracts before amplification [29, 53], it is always advisable to reduce the amount of predator tissue in the DNA extraction process [9]. Extraction of large spiders using the whole specimen with later homogenization and then using a small portion of the DNA extract may not be advisable because of the overabundance of predator DNA in the sample. The amount of spider tissue can be reduced by selecting one portion of the spider body (legs, prosoma or opisthosoma) and removing body parts that do not include digestive tissue (chelicerae or final segments of legs). A method allowing the enrichment of prey DNA from extractions of predators has been recently described by Krehenwinkel *et al*. [74], in which prey DNA is separated from predator DNA by size selection (high molecular weight correspond to predator DNA while shorter fragments correspond to degraded prey DNA) using a purification step with AMPure XP beads. This latter method is a promising technique to enrich prey DNA by removing predator DNA in gut content analyses and it might have broad utility for use in spiders where it is not feasible to dissect the gut.

Although suboptimal, in some situations the use of legs of medium to large sized spiders, instead of the opisthosoma, may be advisable. Recent studies have revealed that it is possible to detect prey DNA in spiders using non invasive methods such as analysing faecal samples [20] or extracting prey DNA from spiders web [77]. It has also been shown that leg autotomy does not compromise the survival of adult spiders (at least in Mygalomorphae) [78] and there are few apparent fitness costs [79]. In juvenile spiders, lost appendages are even replaced after moulting [25, 80]. Therefore, the utilization of legs (better used for detecting predation with specific primers, or at short time periods after feeding when using metabarcoding) instead of opisthosoma or carapaces, provide an additional non-lethal prey DNA source that may complement the use of other less invasive techniques. These could be most relevant for studying rare or protected spiders. Additionally, the use of legs may be preferred in cases where it is important to minimise damage to the voucher (e.g. unique specimens) or preserve relevant taxonomic characters such as female genitalia or spinnerets.

Considering the metabarcoding analyses, prey DNA recovery in the three samples tested varied between *E*. *caelata*, with zero reads generated, and *T*. *molitor*, with 411 reads generated. The difference in time from the ingestion of prey (*E*. *caelata* 72 h, *T*. *molitor* 65 h, chaser prey consumed after target prey) could be one explanation for the unsuccessful prey recovery of *E*. *caelata*, since longer periods after feeding reduce the detectability of prey DNA [52]. An alternative explanation would be a significant primer bias when amplifying different prey, which would severely affect detectability, favouring the amplification of Coleoptera over Isopoda, as has been reported in previous metabarcoding studies with similar *cox1* primers [74]. On the other hand, in the control sample that combined spiders at shorter times after feeding (t = 0–48 h) (higher amount of prey DNA in the gut), *E*. *caelata* was readily amplified with the universal primers used in this study. Hence a combination of factors (time after feeding and possible primer bias) might be affecting prey detectability.

Another complication inherent to metabarcoding analyses is the probability of having false positives, in which prey that were not consumed by the predator are detected due to contaminations, amplification or sequencing errors, including tag switching [81, 82]. In our study the presence of false positives seems unlikely, because the spiders used were feed with known taxa, although some precautions should be taken in standard environmental DNA metabarcoding (eDNA) or molecular prey detection studies to avoid contaminations during the different steps of sampling, lab protocols and bioinformatics analyses, especially if samples from other studies are multiplexed within the same sequencing run (see Ficetola *et al*. [83] for more details).

In our study, the time after feeding at which half predators tested positive for prey DNA using PCR with specific primers approach was 83 h, which is within the typical range observed for spiders (see Greenstone [42]). Studies conducted on spiders have revealed a broad range of detectability half-lives, from less than 10 h (e.g. Lycosidae fed with one aphid [84] or one springtail [85], to more than 100 h (Tetragnathidae fed with one mirid [86]) (see Greenstone [42] for further details).

Although focused in one particular species, our findings might be applicable cautiously, to species other than *Dysdera* since it has been shown that prey DNA detection success is similar within taxonomically related species [87], but also to similar body sized spiders. It has to be considered that prey detectability half-lives in molecular gut content studies might be affected by the predator/prey combination, thus different prey items for the same predator could have different detection half-lives [88, 89]. It is important to mention that considering all the many factors influencing detection half-lives, each study should adapt conditions to the studied organism and system.

Although there are an increasing number of studies that use metabarcoding and metagenomics to study diet [74, 90–93], only two, Paula *et al*. [91] and Srivathsan *et al*. [90], have estimated the range of prey decay, and thus there is a lack of studies that compare the relative efficiency of specific primer approaches versus high throughput sequencing approaches, so more studies in this topic are needed. Paula *et al*. [91], using PCR-free direct shotgun sequencing, found similar decay rates to previous studies using PCR based methods and in some cases, metagenomic sequencing appears to enable prey detection for longer.

Although in our study we have demonstrated that all parts (legs, prosoma and opisthosoma) of the medium-sized spider, *D*. *verneaui*, are suitable for prey DNA detection when using PCR with specific primers, we further recommend using the opisthosoma to amplify gut content after long periods after feeding, in agreement with previous studies [74]. We also suggest using metabarcoding with caution in these studies due to differential amplification of DNA from different groups. Employing metabarcoding approaches with verification studies using specific primers may clarify these feeding interactions.

## Supporting information

S1 Table List of target and non-target prey used for specific primer design, indicating the *cox1* primers used to amplify them (see also Table 1). GenBank accession numbers of sequences downloaded (-), plus the new sequences generated in this study.
(DOCX)

S2 Table List of non-target prey tested against the specific primer designed for *E*. *caelata*. Primers used to amplify *cox1* to test for extraction success.
(DOCX)

S1 Fig DNA decay rate curves in the three body part tested in *D*. *verneaui*. Lines are fitted probit models with 83% fiducial confidence limits (dashed lines).
(TIF)
