# Supplementary material for: Molecular gut content analysis of different spider body parts
Source: PLoS One. 2018 May 30;13(5):e0196589. doi: 10.1371/journal.pone.0196589 (PMC5976152; doi:10.1371/journal.pone.0196589)
Supplement: S1 Table — GenBank accession numbers of sequences downloaded (-), plus the new sequences generated in this study. (DOCX) [file pone.0196589.s001.docx]

**Table 1. Supporting information**

List of target and non-target prey used for specific primer design, indicating the *cox1* primers used to amplify them (see also Table 1). GenBank accession numbers of sequences downloaded (-), plus the new sequences generated in this study.

| **Code** | **Species** | **Primers used**  **to amplify** | **GenBank**  **Accession number** |
| --- | --- | --- | --- |
| DY-009 | *Porcellio* sp. | (1-2) (3-4) | MH279706 |
| DY-010 | *Porcellio* sp. | (1-2) (3-4) | MH279707 |
| DY-011 | *Porcellio* sp. | (1-2) (3-4) | MH279708 |
| DY-013 | *Eluma caelata* | (1-2) | MH279709 |
| DY-077 | *Armadillidium vulgare* | (1-2) (3-4) | MH279710 |
| DY-079 | *Porcellio* sp. | (1-2) (3-4) | MH279711 |
| DY-080 | *Porcellio* sp. | (1-2) (3-4) | MH279712 |
| DY-083 | *Porcellio* sp. | (1-2) (3-4) | MH279713 |
| DY-081 | *Porcellio* sp. | (1-2) (3-4) | MH279714 |
| DY-085 | *Porcellio* sp. | (1-2) (3-4) | MH279715 |
| DY-086 | *Porcellio* sp. | (1-2) (3-4) | MH279716 |
| DY-187 | *Dilta* sp. | (1-2) (3-4) | MH279717 |
| DY-190 | *Dilta* sp. | (1-2) (3-4) | MH279718 |
| DY-191 | *Neoasterolepisma* | (1-2) (3-4) | MH279719 |
| DY-192 | *Neoasterolepisma* | (1-2) (3-4) | MH279720 |
| DY-194 | *Stygnocoris subglaber* | (1-2) (3-4) | MH279721 |
| DY-196 | *Stygnocoris subglaber* | (1-2) (3-4) | MH279722 |
| DY-197 | *Polydesmus coriaceus* | (1-2) (3-4) | MH279723 |
| DY-198 | *Polydesmus coriaceus* | (1-2) (3-4) | MH279724 |
| DY-199 | *Orchestia* sp. | (1-2) | MH279725 |
| DY-200 | *Orchestia* sp. | (1-2) | MH279726 |
| DY-201 | *Bunochelis spinifera* | (1-2) (3-4) | MH279727 |
| DY-202 | *Bunochelis spinifera* | (1-2) (3-4) | MH279728 |
| DY-203 | *Bunochelis spinifera* | (3-4) | MH279729 |
| DY-204 | *Bunochelis spinifera* | (1-2) (3-4) | MH279730 |
| DY-205 | *Cymindis velata* | (1-2) (3-4) | MH279731 |
| DY-206 | *Cymindis velata* | (1-2) (3-4) | MH279732 |
| DY-209 | *Cymindis simillima* | (1-2) | MH279733 |
| DY-210 | *Cymindis simillima* | (1-2) | MH279734 |
| DY-211 | *Ocypus* sp. | (1-2) (3-4) | MH279735 |
| DY-213 | *Ommatoiulus moreletii* | (1-2) | MH279736 |
| DY-215 | *Phyllodromica brullei* | (1-2) (3-4) | MH279737 |
| DY-216 | *Phyllodromica sp.* | (1-2) (3-4) | MH279738 |
| DY-234 | *Porcellio* sp. | (1-2) (3-4) | MH279739 |
| DY-235 | *Porcellio* sp. | (1-2) (3-4) | MH279740 |
| DY-236 | *Porcellio* sp. | (1-2) (3-4) | MH279741 |
| DY-237 | *Porcellio* sp. | (1-2) (3-4) | MH279742 |
| DY-238 | *Porcellio* sp. | (1-2) (3-4) | MH279743 |
| **-** | *Opilio parietinus* | - | AF370832.1 |
| **-** | *Dilta littoralis* | - | AF370847.1 |
| **-** | *Phyllodromica subaptera* | - | AM600684.1 |
| **-** | *Phyllodromica iberica* | - | AM600690.1 |
| **-** | *Phyllodromica quadracantha* | - | AM600692.1 |
| **-** | *Porcellio scaber* | - | DQ305142.1 |
| **-** | *Armadillidium* sp. | - | DQ889079.1 |
| **-** | *Porcellio spinicornis* | - | DQ889123.1 |
| **-** | *Armadillidium lobocurvum* | - | EF027714.1 |
| **-** | *Armadillidium lobocurvum* | - | EF027715.1 |
| **-** | *Armadillidium lobocurvum* | - | EF027716.1 |
| **-** | *Armadillidium nasatum* | - | FN824097.1 |
| **-** | *Armadillidium nasatum* | - | FN824098.1 |
| **-** | *Armadillidium nasatum* | - | FN824099.1 |
| **-** | *Armadillidium vulgare* | - | FN824103.1 |
| **-** | *Armadillidium vulgare* | - | FN824104.1 |
| **-** | *Armadillidium vulgare* | - | FN824105.1 |
| **-** | *Porcellio baidensis* | - | FN824111.1 |
| **-** | *Porcellio baidensis* | - | FN824112.1 |
| **-** | *Porcellio baidensis* | - | FN824114.1 |
| **-** | *Porcellio siculoccidentalis* | - | FN824116.1 |
| **-** | *Porcellio hyblaeus* | - | FN824117.1 |
| **-** | *Porcellio hyblaeus* | - | FN824118.1 |
| **-** | *Porcellio laevis* | - | FN824119.1 |
| **-** | *Porcellio laevis* | - | FN824120.1 |
| **-** | *Porcellio laevis* | - | FN824121.1 |
| **-** | *Porcellio laevis* | - | FN824122.1 |
| **-** | *Porcellio laevis* | - | FN824123.1 |
| **-** | *Porcellio imbutus* | - | FN824125.1 |
| **-** | *Porcellio imbutus* | - | FN824126.1 |
| **-** | *Porcellio imbutus* | - | FN824127.1 |
| **-** | *Porcellio baidensis* | - | FN824128.1 |
| **-** | *Porcellionides pruinosus* | - | FN824135.1 |
| **-** | *Porcellionides pruinosus* | - | FN824136.1 |
| **-** | *Porcellionides pruinosus* | - | FN824137.1 |
| **-** | *Porcellionides pruinosus* | - | FN824138.1 |
| **-** | *Porcellionides pruinosus* | - | FN824139.1 |
| **-** | *Porcellionides pruinosus* | - | FN824140.1 |
| **-** | *Stygnocoris rusticus* | - | HQ106406.1 |
| **-** | *Stygnocoris rusticus* | - | HQ106407.1 |
| **-** | *Stygnocoris rusticus* | - | HQ106408.1 |
| **-** | *Stygnocoris sabulosus* | - | HQ106414.1 |
| **-** | *Stygnocoris sabulosus* | - | HQ106415.1 |
| **-** | *Stygnocoris sabulosus* | - | HQ978681.1 |
| **-** | *Polydesmus* sp. | - | HQ979247.1 |
| **-** | *Phalangium opilio* | - | HQ979250.1 |
| **-** | *Ommatoiulus rutilans* | - | JQ350459.1 |
| **-** | *Polydesmus denticulatus* | - | JQ350460.1 |
| **-** | *Polydesmus* cf. | - | JQ350461.1 |
| **-** | *Polydesmus monticola* | - | JQ350462.1 |
| **-** | *Orchestia guancha* | - | JX094882.1 |
| **-** | *Cymindis cribricollis* | - | JX260002.1 |
| **-** | *Cymindis neglectus* | - | JX260014.1 |
| **-** | *Cymindis unicolor* | - | JX260021.1 |
| **-** | *Cymindis* sp. | - | JX260687.1 |
| **-** | *Cymindis interior* | - | JX260689.1 |
| **-** | *Cymindis planipennis* | - | JX260690.1 |
| **-** | *Dysdera ramblae* | - | AF244311.1 |
| **-** | *Dysdera ramblae* | - | AF244312.1 |
| V170CRC | *Dysdera vernaui* | - | KC785739 |
| V176MAT | *Dysdera vernaui* | - | KC785783 |
| dsi347P | *Dysdera silvatica* | - | HQ396328 |
| dsi362H | *Dysdera silvatica* | - | HQ396329 |
| dsiGk94 | *Dysdera silvatica* | - | AF244273 |
| G371G | *Dysdera gomerensis* | - | KC785792 |
| G374H | *Dysdera gomerensis* | - | KC785789 |
