# Supplementary material for: Molecular gut content analysis of different spider body parts
Source: PLoS One. 2018 May 30;13(5):e0196589. doi: 10.1371/journal.pone.0196589 (PMC5976152; doi:10.1371/journal.pone.0196589)
Supplement: S2 Table — Primers used to amplify cox1 to test for extraction success. (DOCX) [file pone.0196589.s002.docx]

**Table 2. Supporting information**

List of non-target prey tested against the specific primer designed for *E. caelata*. Primers used to amplify *cox1* to test for extraction success.

| **Code** | **Species** | **Family** | **Order** | **Primers used to amplify** |
| --- | --- | --- | --- | --- |
| DY-029 | *Tegenaria pagana* | Agelenidae | Araneae | (1-2) |
| DY-230 | *Macarophaeus varius* | Gnaphosidae | Araneae | (1-2) |
| DY-221 | *Tenuiphantes canariensis* | Linyphiidae | Araneae | (1-2) |
| DY-228 | *Improphantes furcabilis* | Linyphiidae | Araneae | (1-2) |
| DY-229 | cf. *Agyneta canariensis* | Linyphiidae | Araneae | (1-2) |
| DY-070 | *Alopecosa cedroensis* | Lycosidae | Araneae | (3-4) |
| DY-072 | *Alopecosa gomerae* | Lycosidae | Araneae | (3-4) |
| DY-223 | *Zoropsis rufipes* | Lycosidae | Araneae | (1-2) |
| DY-030 | *Cladycnis insignis* | Pisauridae | Araneae | (1-2) |
| DY-071 | *Segestria bavarica* | Segestriidae | Araneae | (3-4) |
| DY-068 | *Steatoda grossa* | Theridiidae | Araneae | (3-4) |
| DY-226 | *Steatoda nobilis* cf | Theridiidae | Araneae | (1-2) |
| DY-224 | *Enoplognatha sattleri* | Theridiidae | Araneae | (1-2) |
| DY-034 | *Calathus arbaxoides* | Carabidae | Coleoptera | (1-2) |
| DY-033 | *Cymindis zargoides* | Carabidae | Coleoptera | (1-2) |
| DY-054 | *Dicrodontus aptinoides* | Carabidae | Coleoptera | (5-6) |
| DY-055 | *Gomerina calathiformis* | Carabidae | Coleoptera | (5-6) |
| DY-057 | *Harpalus schaumi* | Carabidae | Coleoptera | (5-6) |
| DY-053 | *Nesacinopus micans* | Carabidae | Coleoptera | (1-2) |
| DY-052 | *Paraeutrichopus pecoudi* | Carabidae | Coleoptera | (1-2) |
| DY-056 | *Trechus flavocinctus gomerae* | Carabidae | Coleoptera | (5-6) |
| DY-060 | *Coccinella miranda* | Coccinellidae | Coleoptera | (5-6) |
| DY-036 | *Cryptophagus ellipticus* | Cryptophagidae | Coleoptera | (1-2) |
| DY-051 | *Cryptophagus* sp. | Cryptophagidae | Coleoptera | (5-6) |
| DY-046 | *Cryptos* sp. | Cryptophagidae | Coleoptera | (1-2) |
| DY-038 | *Alloplinthus musicus* | Curculionidae | Coleoptera | (1-2) |
| DY-058 | *Echinodera* sp. | Curculionidae | Coleoptera | (5-6) |
| DY-037 | *Laparocerus grossepunctatus* | Curculionidae | Coleoptera | (1-2) |
| DY-039 | *Lichenophagus* sp. | Curculionidae | Coleoptera | (1-2) |
| DY-050 | *Cardiophorus gomerensis* | Elateridae | Coleoptera | (5-6) |
| DY-041 | *Cardiophorus* sp. | Elateridae | Coleoptera | (1-2) |
| DY-064 | *Meloe* sp. | Meloidae | Coleoptera | (5-6) |
| DY-040 | *Ocypus* sp. | Staphylinidae | Coleoptera | (5-6) |
| DY-062 | *Ocypus* sp. | Staphylinidae | Coleoptera | (5-6) |
| DY-061 | *Ocypus sylvaticus* | Staphylinidae | Coleoptera | (5-6) |
| DY-063 | *Nesotes* sp. | Tenebrionidae | Coleoptera | (1-2) (3-4) (5-6) |
| DY-035 | *Tarphius caudatus* | Zopheridae | Coleoptera | (5-6) |
| DY-059 | *Tarphius humerosus* | Zopheridae | Coleoptera | (5-6) |
| DY-042 | *Guanchia* sp. | Forficulidae | Dermaptera | (3-4) |
| DY-065 | *Guanchia storai* | Forficulidae | Dermaptera | (5-6) |
| DY-092 | Unknown species | *Chloropidae* | Diptera | (5-6) |
| DY-094 | *Suillia oceana* | Heleomyzidae | Diptera | (5-6) |
| DY-096 | Unknown species | *Hybotidae* | Diptera | (5-6) |
| DY-095 | Unknown species | *Phoridae* | Diptera | (3-4) |
| DY-088 | Unknown species | *Sciaridae* | Diptera | (3-4) |
| DY-093 | *Melanostoma incompletum* | Syrphidae | Diptera | (3-4) |
| DY-066 | *Irwiniella frontata* | Therevidae | Diptera | (5-6) |
| DY-044 | Unknown species |  | *Geophilomorpha* | (1-2) |
| DY-217 | Unknown species | *Lumbricidae* | Haplotaxida | (1-2) |
| DY-091 | Unknown species | *Cixiidae* | Hemiptera | (1-2) |
| DY-043 | *Eremocoris maderensis* | Lygaeidae | Hemiptera | (1-2) |
| DY-089 | *Ploiaria chilensis* | Reduviidae | Hemiptera | (5-6) |
| DY-090 | Unknown species |  | Hemiptera (Homoptera) | (3-4) |
| DY-067 | *Pimpla* sp. | Ichneumonidae | Hymenoptera | (5-6) |
| DY-087 | *Pimpla* sp. | Ichneumonidae | Hymenoptera | (5-6) |
| DY-218 | *Dolichoiulus* sp. | Julidae | Julida | (1-2) |
| DY-031 | *Ascotis fortunata* | Geometridae | Lepidoptera | (1-2) (3-4) (5-6) |
| DY-032 | Unknown species (larvae) |  | Lepidoptera | (1-2) |
| DY-045 | *Lithobius* sp. | Lithobiidae | Lithobiomorpha | (1-2) (3-4) |
| DY-048 | *Hirudicryptus canariensis* | Siphonocryptidae | Polyzoniida | (1-2) |
| DY-220 |  |  | Class Gastropoda | (1-2) |
| DY-219 |  |  | Phyllum Nematoda | (1-2) |
|  |  |  |  |  |
