# Supplementary figures and images for: Molecular gut content analysis of different spider body parts
Source: PLoS One. 2018 May 30;13(5):e0196589. doi: 10.1371/journal.pone.0196589 (PMC5976152; doi:10.1371/journal.pone.0196589)

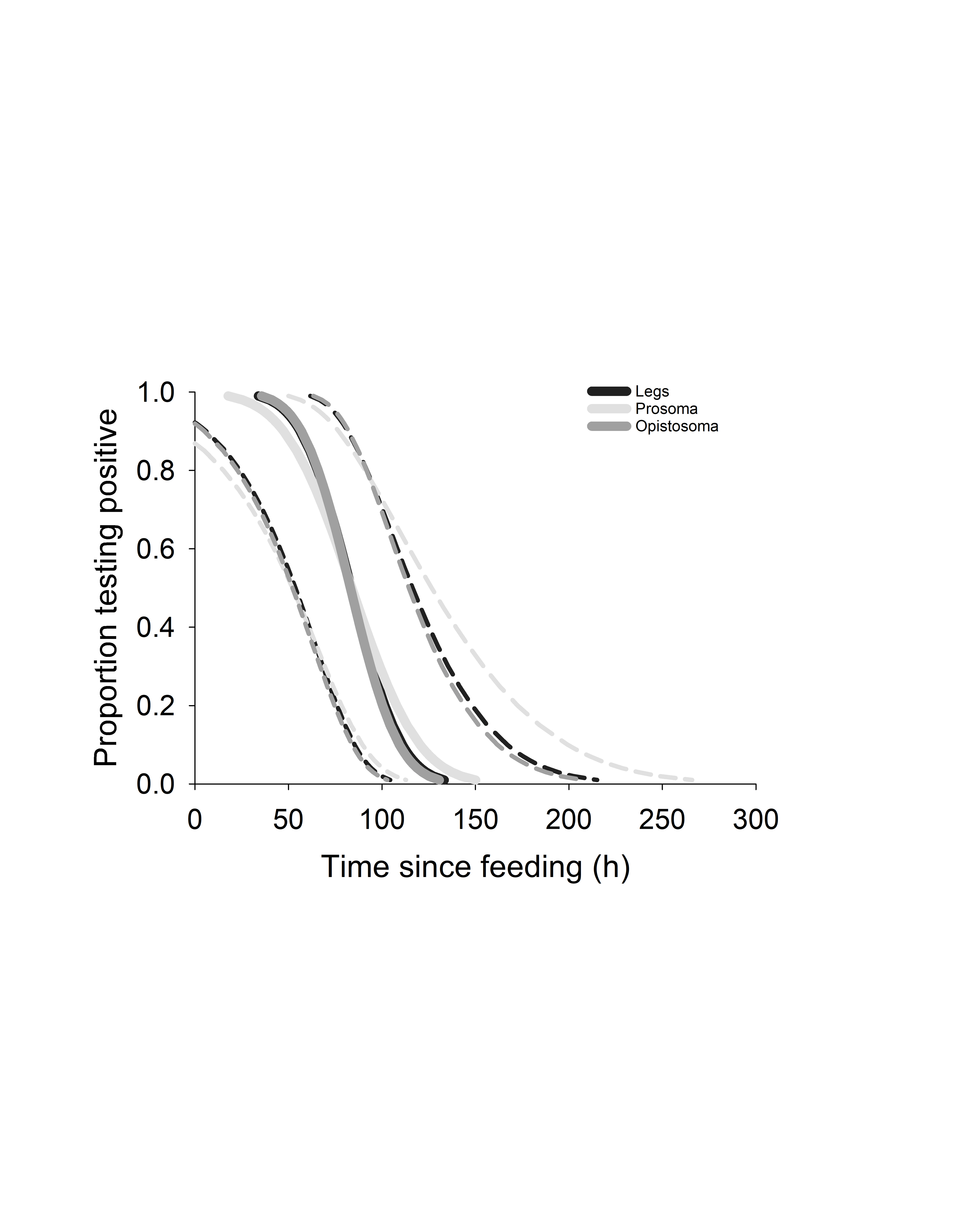

Supplement: S1 Fig — Lines are fitted probit models with 83% fiducial confidence limits (dashed lines). (TIF) [file pone.0196589.s003.tif]
